# Pkd2 affects the architecture of zebrafish left-right organizer

**DOI:** 10.1186/2046-2530-4-S1-P84

**Published:** 2015-07-13

**Authors:** R Jacinto, P Sampaio, M Roxo-Rosa, S Lopes

**Affiliations:** 1 CEDOC, NOVA Medical School, Lisbon, Portugal

## Background

Dorsal anterior clustering (DAC) of motile cilia in the left-right organizer (LRO) is crucial for normal fluid flow dynamics and correct laterality in zebrafish [1]. We directly demonstrated that *charon*/*dand5 *transcription is negatively regulated by strong flow in zebrafish LRO [1] which suggests that LRO cells have the ability to sense fluid flow and influence gene expression patterns, but how? Pkd2 ion channel is a good candidate because it participates in a mechanosensory complex that senses fluid flow and induces a calcium inward flux in kidney cells [2] and in nodal cells [3]. In agreement, mouse and zebrafish mutants for Pkd2 have LR defects [4,5]. However, Pkd2 is also involved in cell polarity during migration [6] and in extracellular matrix deposition [7] implying a role for Pkd2 in cell morphogenesis.

## Objective

Determine whether Pkd2 knockdown affects DAC of ciliated LRO cells.

## Methods

*dnah7 *morpholino [1] was injected to generate static cilia without affecting DAC [1]. Each embryo was screened for static cilia by high-speed videomicroscopy. In parallel, Pkd2 knockdowns were imaged for flow dynamics followed by quantification of anterior / posterior cilia number through two-photon microscopy.

## Results

Pkd2 knockdown, contrary to Dnah7 knockdown, caused a remodelling in the LRO architecture by disrupting DAC. Moreover, Pkd2 knockdown resulted in abnormal fluid flow as a consequence of defective DAC.

## Conclusions

Comparing Dnah7 and Pkd2 knockdowns we concluded that Pkd2 mediated pathway affects LRO morphogenesis by a mechanism that seems to be independent of its role in fluid flow mechanosensation. Meaning that Pkd2 triggers additional responses from those caused by LRO flow.

Supported by FCT-ANR/BEX-BID/0153/2012 grant.
